# Insider's Guide to Quality, Affordable Healthcare: Practical Strategies to Navigate Our Complex System and Save Money

**DOI:** 10.5195/jmla.2020.960

**Published:** 2020-07-01

**Authors:** Paige Scudder

**Affiliations:** 1 paige.n.scudder@dartmouth.edu, Research and Education Librarian, Biomedical Libraries, Dartmouth College, Hanover, NH

A Google search of “navigating healthcare system” yields over a million results; a search of “healthcare help” provides even more results. In a world where answers are at the whim of an algorithm, where more information often leads to more questions, the book, *Insider's Guide to Quality, Affordable Healthcare*: *Practical Strategies to Navigate Our Complex System and Save Money*, takes the reader on a thoughtfully planned and systematic journey through the US healthcare system.

Medical doctors Lawrence Lazarus and Jeffery Foster divide their book into four sections: “Dealing with Major Changes in Healthcare,” “Finding the Best Healthcare Providers,” “Navigating Specific Healthcare Treatments,” and “Further Healthcare Evaluations and Choices.” Each section contains three to four chapters, but, more notably, each chapter begins with a description of what will be covered and ends with a bulleted summary of what was discussed. This organizational technique makes it easy to follow along if you are reading the book cover to cover, if you are jumping around between chapters, or if you are utilizing the book as a reference.

The first section, “Dealing with Major Changes in Healthcare,” focuses on ways you, as a patient, can advocate for yourself and your loved ones. For example, how you can compensate for briefer doctor visits, how you can reduce your risk of medical errors, and how you can save money. Most notably, one of the authors discusses that the “greatest gift you can bestow upon your children and grandchildren” is having a healthy lifestyle and practicing good strategies for securing healthcare.

One of the authors developed tinnitus (ringing in the ears) when he turned sixty-five, about the age that his father and older sister developed the same condition. Consulting several ear specialists, he learned that there was no effective treatment. But by doing some investigation, he learned about preventative measures that may help his children avoid inheriting tinnitus, such as avoiding exposure to loud sounds (e.g., rock concerts) and avoiding teeth grinding. These preventative measures were shared with his adult children, who were appreciative and agreed to share the information with their children (p. 20).

By sharing personal stories and using clinical examples throughout the book, the authors illustrate the importance of making health concerns a conversation rather than a topic to be avoided. They also illustrate how working with doctors to learn about a personal health problem benefits the entire family, providing them with new information that may prevent or mitigate their own future health problems.

The second section, “Finding the Best Healthcare Providers,” shares advice on finding the best “captain or coordinator of your healthcare team” and how a good primary care physician can make all the difference in your healthcare. While the authors describe the healing side and the business side, they also compile multiple lists of questions to consider when selecting a doctor, as well as suggestions on how to make the most of every visit. These chapters are designed to empower you to make the best choice and to remind you that who you choose as your primary care physicians is just that, a choice.

The section, “Navigating Specific Healthcare Treatments,” continues the theme of the importance of having a healthcare plan. These chapters provide great examples and offer a helpful key point summary at the end. This summary provides a neat list you can use to prepare for a safe hospital stay, to receive the best care during your stay, and prepare questions about your prescriptions as well as preparations to make when returning home.

The final section, “Further Healthcare Evaluations and Choices,” focuses on choosing the best insurance, your rights as a patient, alternative medicine, and the future of healthcare. This section neatly wraps up the authors' message of how important it is to take control of your health, your healthcare, and everything in between.

The US healthcare system can seem daunting and complicated to both the general public and to providers working in it. The *Insider's Guide to Quality, Affordable Healthcare* provides concrete examples you can relate to with a conversational tone that feels as though the authors are sitting around a table with you as they provide practical advice. Whether you are a physician looking to navigate your patient through the system or a patient filled with questions, this book is an excellent reference to get you started, as well as a selection of curated resources for further reading.

